# Serine protease inhibitors protect better than IL-10 and TGF-β anti-inflammatory cytokines against mouse colitis when delivered by recombinant lactococci

**DOI:** 10.1186/s12934-015-0198-4

**Published:** 2015-02-26

**Authors:** Luis G Bermúdez-Humarán, Jean-Paul Motta, Camille Aubry, Pascale Kharrat, Laurence Rous-Martin, Jean-Michel Sallenave, Céline Deraison, Nathalie Vergnolle, Philippe Langella

**Affiliations:** INRA, Commensal and Probiotics-Host Interactions Laboratory, UMR 1319 Micalis, F-78350 Jouy-en-Josas, France; AgroParisTech, UMR1319 Micalis, F-78350 Jouy-en-Josas, France; Inserm, U1043, Toulouse, F-31300 France; CNRS, U5282, Toulouse, F-31300 France; Université de Toulouse, UPS, Centre de Physiopathologie de Toulouse Purpan (CPTP), Toulouse, F-31300 France; Department of Biological Sciences, Faculty of Science, University of Calgary, Calgary, Alberta Canada; INSERM U874, Institut Pasteur, 25 rue du Dr Roux, 75015 Paris, France; INSERM U1152, Faculté de Médecine site Bichat, Université Paris Diderot, 16, rue Henri Huchard, 75018 Paris, France; Université Sorbonne Paris Cité, Université Paris Diderot, rue du Dr Roux, 75015 Paris, France

**Keywords:** Elafin, Proteases, Inflammation, Colitis, Inflammatory bowel disease, *Lactococcus lactis*, Probiotics

## Abstract

**Background:**

Different studies have described the successful use of recombinant lactic acid bacteria (recLAB) to deliver anti-inflammatory molecules at the mucosal level to treat Inflammatory Bowel Disease (IBD).

**Methods:**

In order to identify the best strategy to treat IBD using recLAB, we compared the efficacy of different recombinant strains of *Lactococcus lactis* (the model LAB) secreting two types of anti-inflammatory molecules: cytokines (IL-10 and TGF-β1) and serine protease inhibitors (Elafin and Secretory Leukocyte Protease Inhibitor: SLPI), using a dextran sulfate sodium (DSS)-induced mouse model of colitis.

**Results:**

Our results show that oral administration of recombinant *L. lactis* strains expressing either IL-10 or TGF-β1 display moderate anti-inflammatory effects in inflamed mice and only for some clinical parameters. In contrast, delivery of either serine protease inhibitors Elafin or SLPI by recLAB led to a significant reduction of intestinal inflammation for all clinical parameters tested. Since the best results were obtained with Elafin-producing *L. lactis* strain, we then tried to enhance Elafin expression and hence its delivery rate by producing it in a *L. lactis* mutant strain inactivated in its major housekeeping protease, HtrA. Strikingly, a higher reduction of intestinal inflammation in DSS-treated mice was observed with the Elafin-overproducing *htrA* strain suggesting a dose-dependent Elafin effect.

**Conclusions:**

Altogether, these results strongly suggest that serine protease inhibitors are the most efficient anti-inflammatory molecules to be delivered by recLAB at the mucosal level for IBD treatment.

## Background

Inflammatory bowel disease (IBD) is a group of chronic inflammatory disorders that cause inflammation of the digestive tract. The two major forms of IBD are Crohn’s Disease (CD) and Ulcerative Colitis (UC) and they are characterized by an uncontrolled inflammatory response to lumenal content [1]. Despite the fact that several anti-inflammatory molecules have been tested in preclinical and clinical models for IBD treatment, their therapeutic potential and clinical application have been frequently hampered by different obstacles such as successful delivery or even toxic side-effects [1]. In the last 10 years, an increasing number of clinical and experimental studies have proven that probiotic bacteria may counteract the chronic inflammatory process. This effect is achieved by stabilizing the gut microbial environment and permeability barrier functions and by modulating the microbiota composition [2,3]. In addition, the successful use of food-grade Lactic Acid Bacteria (LAB) for the oral delivery of anti-inflammatory molecules to the inflamed intestine in preclinical experiments, as well as clinical trials have been reported [4-10]. This approach is based on the local synthesis and delivery of therapeutic molecules by viable recombinant LAB (recLAB) *in situ*.

The pioneer use of such recLAB for the prevention and treatment of experimental IBD was performed by Steidler *et al.* [11] who developed a recombinant strain of *Lactococcus lactis* (the LAB model) secreting biologically active anti-inflammatory cytokine IL-10. Interestingly, the authors showed that daily oral administration of *L. lactis* IL-10 in mice resulted in ~50% reduction in dextran sulfate sodium (DSS)-induced colitis [11]. The beneficial effect of *L. lactis* IL-10 strain was dependent on the *in situ* secretion of IL-10 by recombinant live lactococci. Steidler *et al.* have then developed the first biocontainment system for *L. lactis* IL-10 strain to start the first human clinical study using it [12]. A phase I clinical trial was then conducted with this biocontained *L. lactis* IL-10 strain in Crohn’s disease patients, showing that the containment strategy was effective [13]. Following this, a phase IIA trial was performed and a press release was published in 2009 revealing that all three primary endpoints have been met: i) safety and tolerability; ii) environmental containment and iii) assessment of biomarkers associated with the strain (data from ActoGeniX press release). Unfortunately, the clinical results did not reveal a statistically significant difference in mucosal healing with *L. lactis* IL-10 *versus* placebo. In view of these results, one can wonder whether IL-10 was the right choice of anti-inflammatory molecule to be delivered by recLAB. Other anti-inflammatory molecules to be delivered by recLAB should thus be tested.

Recent work has involved proteases and their endogenous inhibitors in the pathology of IBD [14-16]. Indeed, intestinal tissues from CD and UC patients showed elevated proteolytic activity [14,15]. This high proteolytic activity could be due to either upregulated protease expression, or decreased efficacy or expression of endogenous proteases inhibitors, or both. Transgenic mice producing human Elafin, an endogenous serine protease inhibitor found in the human gut, are protected from colitis in various mouse models of IBD [14]. We thus constructed recombinant *L. lactis* strains able to deliver Elafin at the mucosal level. We showed that the Elafin delivered by these recLAB prevents inflammation, accelerates mucosal healing and restores colon homeostasis in mice [17]. Although Elafin delivery at the mucosal surface by LAB was shown to efficiently reduce inflammatory signs in mouse colitis, one can wonder whether other protease inhibitors with a broader spectrum of inhibition might be as or more efficient. The Secretory Leukocyte Protease Inhibitor (SLPI, another serine protease inhibitor) inhibits the same elastases as Elafin (Elastase and Proteinase-3), but also inhibits Cathepsin G and trypsin, tryptase and chymase, major proteases contained in inflammatory cell granules. SLPI therefore appears as another possible attractive candidate to be delivered by LAB.

Besides the anti-inflammatory cytokine *L. lactis* IL-10, there is also Transforming Growth Factor-β1 (TGF-β) which is an inhibitory cytokine recognized as a key regulator of immunological homeostasis and inflammatory responses [18]. Mice deficient for TGF-β1 expression suffered from a more extensive autoimmune process with inflammatory infiltrates, involving multiple organs, including the intestine [19]. More important, despite the broad anti-inflammatory and immune suppressive actions of TGF-β1, to our knowledge, the potential anti-inflammatory effects of a mucosal delivery of this cytokine have not yet been compared to that of IL-10.

In order to identify the best strategy to treat IBD using recLAB as mucosal delivery carrier, we thus performed a comparison between *L. lactis* strains secreting between cytokines or serine protease inhibitors, using a DSS-induced colitis mouse model. We compared the efficacy of different recombinant strains of *L. lactis* secreting i) either IL-10 or TGF-β1 as anti-inflammatory cytokines, and ii) either Elafin or SLPI as serine protease inhibitors. To further identify the best strategy to use recLAB, we constructed a recLAB strain inactivated in its major extracellular housekeeping protease, the high temperature requirement A (HtrA), which is supposed to produce higher quantities of the desired molecule [20]. Such approach aimed at determining the best vector, and defined whether a dose-dependent effect of the delivered molecule is important.

The most efficient protection against colitis was obtained by treatments with Elafin-producing *L. lactis* strain. This protective effect was enhanced with the Elafin-overproducing *htrA* strain, suggesting a dose-dependent effect of Elafin delivery. Altogether, these results showed that serine protease inhibitors are more efficient than anti-inflammatory cytokines as anti-inflammatory molecules to be delivered by recLAB at the mucosal level for IBD treatment.

## Results

### Production of anti-inflammatory cytokines and serine protease inhibitors by *L. lactis*

**Figure 1:**
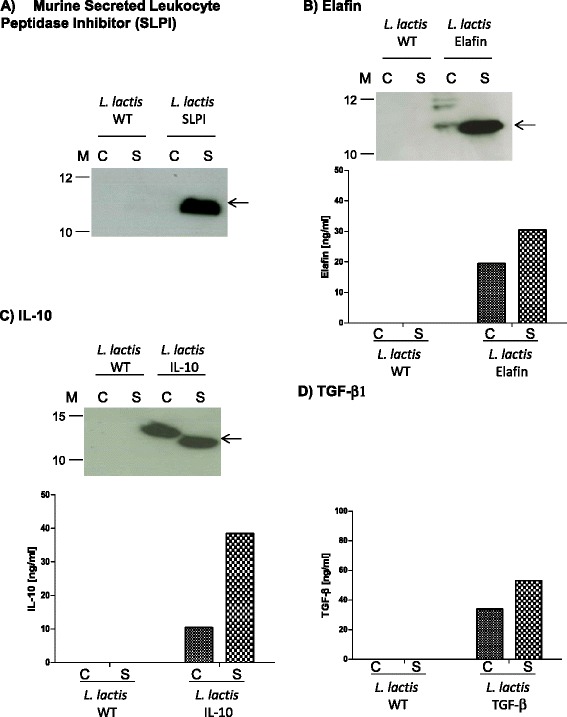
**Characterization of recombinant** ***Lactococcus lactis*** **strains producing either anti-inflammatory molecules (IL-10 and TGF-β1) or protease inhibitors (Elafin and SLPI).** Protein production and secretion were analyzed by Western blotting **(panels A, B and C)** and ELISA **(panel D)** 1 h after induction with 1 ng/ml of nisin, the samples were then harvested at late exponential phase (~OD_600_ = 0.8-1.0). Arrows indicate positions of mature proteins (*eg*. mSLPI **panel A**, human Elafin **panel B**, and mIL-10 **panel C**). Abbreviations: C, cell lysates; S, supernatant fraction; M, positions and sizes of molecular mass markers.

We constructed 4 different recombinant *L. lactis* strains that secrete i) either murine IL-10 (LL-IL-10) or murine TGF-β (LL-TGF-β) as anti-inflammatory cytokines or ii) either human Elafin (*L. lactis* Elafin) or murine SLPI (*L. lactis* SLPI) as serine protease inhibitors. Production and secretion of these molecules were evaluated, and quantified when possible, by Western blot and ELISA assays, respectively. As shown in Figure 1A, a clear band was detected in the supernatant (S) fraction from induced LL-SLPI cultures at the expected size for mature SLPI (*ie.* without the SP_Usp45_); in these experimental conditions, no signal was detected in the cell fraction (C) fraction suggesting an optimal secretion efficiency (~100%). Similar analysis of LL-Elafin strain resulted in different profiles in C and S samples (Figure 1B): i) two weak bands in the C fraction which likely correspond to either anomalous Elafin migration on SDS-PAGE or Elafin dimers and a weak band which slightly migrated higher than Elafin and which corresponds to SP_Usp45_-Elafin precursor (preElafin) and ii) a clear single band in the S fraction at the expected size for mature Elafin. In addition, Elafin production and secretion by recombinant lactococci were quantified by ELISA and estimated at ~35 ng/ml (Figure 1B).

Concerning IL-10 expression by LL-IL-10, our results reveal a good production and secretion of this cytokine by recombinant lactococci since more than 70% was found in S fraction (Figure 1C). These results were also validated by ELISA and estimated at 40 ng/ml (Figure 1C).

TGF-β production by LL*-*TGF-β strain was assessed only by ELISA and estimated at 50 ng/ml (Figure 1D). The results show that *L. lactis* is able to produce and efficiently secrete (secretion rate of ~50%) this cytokine.

The quantity of all recombinant molecules secreted from LAB was more or less in the same order of magnitude, insuring thereby the possibility to compare the efficiency of treatments with the different recLAB.

We should mention that none of the recombinant proteins was detected in samples obtained from *L. lactis* wild-type (WT) strain used as the negative control for Western Blot and ELISA assays (Figure 1A-D).

### Serine protease inhibitors-expressing *L. lactis* strains markedly reduced intestinal inflammation in a DSS-induced murine colitis model

To define the best strategy to treat IBD using recLAB, we compared recombinant strains of *L. lactis* secreting the different anti-inflammatory candidates, using a DSS-induced murine colitis model.

**Figure 2:**
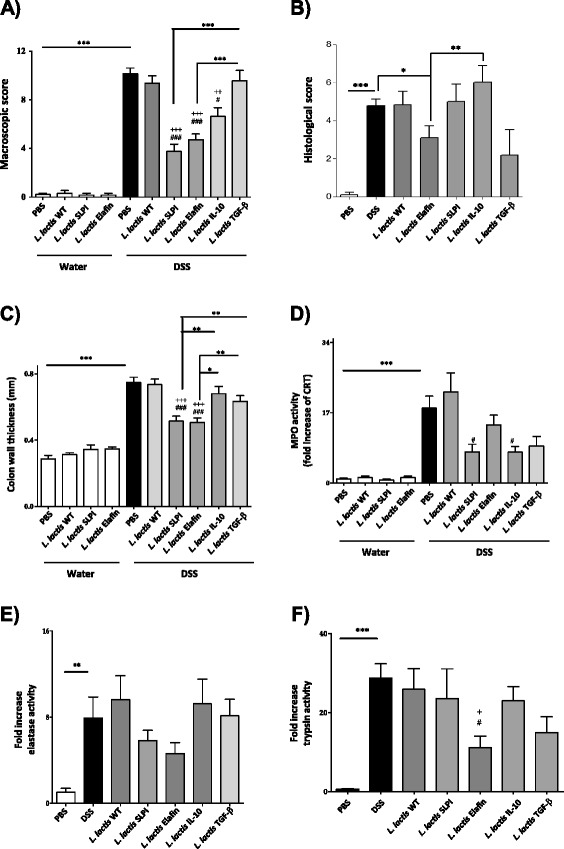
**Effects of recombinant lactococci expressing 4 different molecules in a DSS-induced murine colitis model.** C57BL/6 mice were given water or water containing 5% DSS for 7 days and then received daily oral treatments for 7 days with either, vehicle (PBS), *L. lactis* wild-type (WT), Elafin-, SLPI-, IL-10- or TGF-β-expressing strains of *L. lactis*. (A-F). The macroscopic damage **(A)** and microscopic scores **(B)**, colon wall thickness **(C)**, and MPO activity **(D)** in mouse colon tissue are shown. Elastolytic **(E)** and trypsinolytic **(F)** activities were assessed in colonic lumenal washes. Significant differences is *,+,# for *p <* 0.05 , **,++,## for *p <* 0.01 ***,+++,### for *p <* 0.001. + represents significant difference versus DSS and # represents significant difference versus DSS + *L.lactis* WT. Data represents cumulative results of 4 independent experiments of *n =* 6-8 mice per groups.

**Figure 3:**
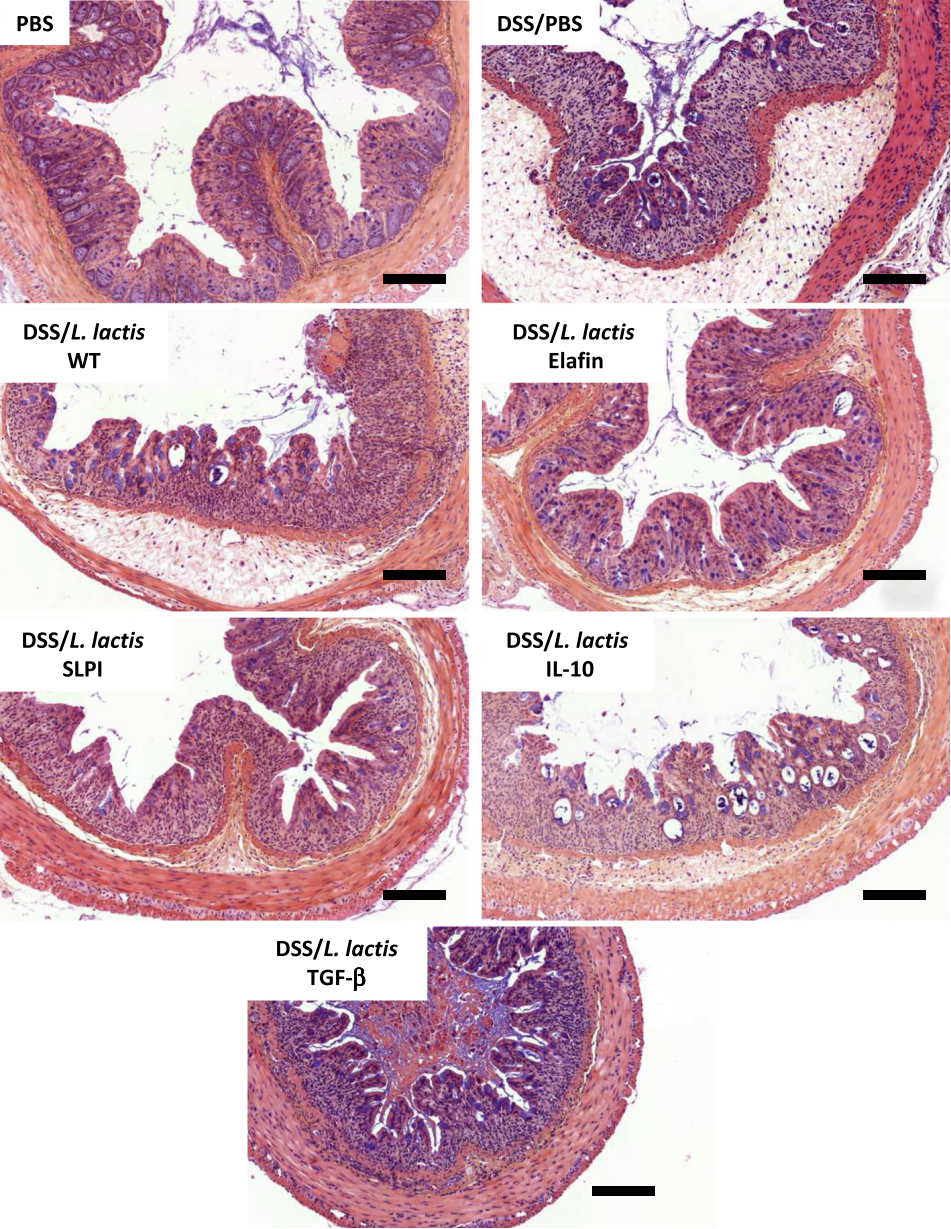
**Representative images of H & E staining on paraffin-wax-embedded sections of the colon of mice are presented.** Scale bars represent 100 μm.

Colitis was induced in C57BL/6 mice by addition of DSS (5% w/v) in drinking water for 7 days. This caused an acute inflammation characterized by increased macroscopic (Figure 2A) and microscopic (Figures 2B and 3) damage score, increased colon thickness (Figure 2C), prominent granulocyte infiltration (MPO activity, Figure 2D) and higher proteolytic activity (Figure 2E and F) in inflamed colonic tissues. Daily oral administrations of recombinant *L. lactis* secreting serine protease inhibitors resulted in a significant reduction of inflammation (decreased macroscopic and microscopic colonic damage scores, colon thickness and MPO activities), when compared to control mice (treated with either PBS or WT *L. lactis* strain). In addition, lower elastolytic activity was observed in colonic washes from mice receiving both LL-Elafin and LL-SLPI (Figure 2E). Trypsin-like activity was also significantly reduced in colonic washes from colitic mice that have received oral treatment with LL-Elafin (Figure 2F). Decreased inflammatory signs (damages score, wall thickness and MPO activity) were also observed in colonic tissues harvested from DSS-treated mice treated with *L. lactis* IL-10 (Figures 2A, B and D and 3). Interestingly, the protective effects against colitis were higher in mice treated with the two *L. lactis* strains recombinant for serine protease inhibitors, than those treated with LL-IL-10 (macroscopic and microscopic score and colon thickness) (Figures 2A and B and 3).

Recombinant *L. lactis* strains expressing TGF-β displayed beneficial effects for MPO activities (Figure 2D) and histological scores (Figure 2B). However, this last, was not significantly different when compared to control mice. These results suggest that TGF-β is a less efficient anti-inflammatory molecule when delivered by recombinant *L. lactis* to treat colitis.

### Improving the anti-inflammatory properties of Elafin-producing *L. lactis*

**Figure 4:**
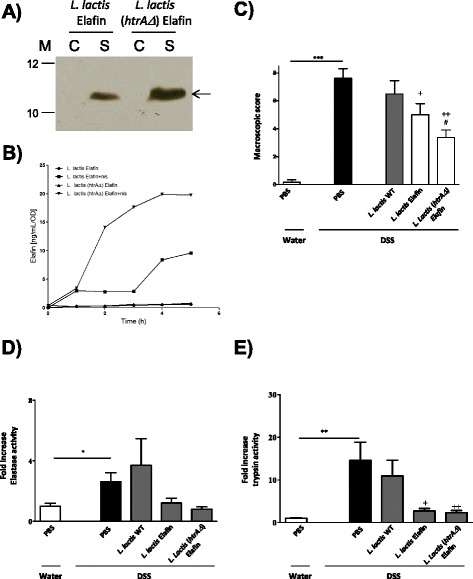
**Enhancing Elafin production by** ***L*** **.** ***lactis*** **htrA mutant strain, as determined by Western blot (A) and ELISA (B) experiments, led to higher protective effects in a DSS-induced murine colitis model.** C57BL/6 mice (*n =* 8 in each group) were given water or water containing 5% DSS for 7 days and then received daily oral treatments for 7 days with either, vehicle (PBS), *L. lactis* WT, Elafin-expressing *L. lactis* (*L. lactis* Elafin) or Elafin-expressing *L. lactis-htra* (*L. lactis* Elafin+). The macroscopic damage score **(C)** of the colon is shown. Elastolytic **(D)** and trypsinolytic **(E)** activities were assessed in colonic lumenal washes. Significant differences is * for *p <* 0.05 , ** for *p <* 0.01 *** for *p <* 0.001.

As oral administration of serine protease inhibitors-producing *L. lactis* strains, and more particularly LL-Elafin, was the most efficient strategy to inhibit DSS-induced colitis, we thus evaluated whether an improvement in the Elafin production and secretion by *L. lactis* could have an impact on its beneficial effects. We have previously reported that inactivation of the major *L. lactis* housekeeping protease, HtrA, allows high-level production and secretion of heterologous proteins [20,21]. We then established pSEC:hElafin plasmid in *L. lactis htrA* strain [22] to obtain *L. lactis* (*htrAΔ*) Elafin and assessed Elafin production and secretion by Western blot (Figure 4A). In addition, Elafin secretion by *L. lactishtrAΔ* Elafin strain was quantified and compared with that of its counterpart *L. lactis* Elafin by ELISA and estimated at ~55 ng/ml *versus* 37 ng/ml (Figure 4A). Since *L. lactis htrAΔ* strain has tendency to grow slower than WT *L. lactis* strain [21] we decided to compare growth curves (data not shown) of both strains and to determine elafin production per bacteria CFU (ng elafin/CFU/time) (Figure 4B). To compare the amounts of Elafin produced by recombinant *L. lactis* the results were normalized to a standard culture volume of 1 ml of 1.0 OD_600_ unit of culture (*e.g.* OD_600_ = 1 = 5×10^8^ CFU of *L. lactis*). As shown in Figure 4B, time-course experiments by ELISA comparing Elafin production by the two strains revealed that, despite its lower grow *L. lactis htrAΔ* strain produces higher levels of Elafin, compared to its counterpart *L. lactis* Elafin strain. This improvement in Elafin yields confirms the interest of *L. lactis htrA* strain to enhance production and secretion of human Elafin. We then compared the effects of these two strains in the DSS-induced murine colitis model. As shown in Figure 4C, oral administration of mice with *L. lactis* (*htrAΔ*) Elafin strain resulted in a better reduction of colonic damage scores, compared to the *L. lactis* Elafin strain. Treatment of mice with the *L. lactishtrAΔ* Elafin strain was also very efficient to reduce proteolytic activity in inflamed colonic tissues: both elastolytic (Figure 4D) and trypsin-like activity (Figure 4E). Indeed, a better inhibition of colonic washes proteolytic activity was achieved with the *L. lactishtrAΔ* Elafin strain, compared to the LL-Elafin strain (Figure 4C-E). Altogether, these results suggest a dose-dependent effect of human Elafin when delivered *in situ* by recombinant *L. lactis*.

## Methods

### Bacterial strains and culture conditions

*Escherichia coli* TG1 [23] was grown in Luria-Bertani (Difco) medium at 37°C with vigorous agitation and both *Lactococcus lactis* MG1363 [24] and *L. lactis* NZ9000 [25] in M17 medium (Difco) supplemented with 0.5% glucose (GM17) at 30°C. Antibiotics were used as follows: for *E. coli* 100 μg/ml of ampicillin or 15 μg/ml of chloramphenicol and for *L. lactis* 10 μg/ml of chloramphenicol.

### Construction of recombinant LAB strains

Gene encoding for murine TGF-β1 (mTGF-β1) was amplified by PCR from plasmid pORF9-mTGF-β1 (InvivoGen) using primers: forward mTGF-β1 (CCA*ATGCAT*CAGCC CTGGATACCAACTATTGC) and reverse mTGF-β1 (GG *ACTAGT*CCTCAGCTGCACTTG CAGGAGC). Gene encoding for murine secretory leukocyte protease inhibitor (mSLPI) was amplified by PCR from plasmid pDK6-mSLPI [26] using primers: forward mSLPI (CCA*ATGCAT*CAGGCAAAAATGATGCTATCAAAATCG) and reverse mSLPI (GG *ACTAGT*CCTCACATCGGGGGCAGGCAGACTTTCC). PCR products were subcloned into pCR:TOPO (Invitrogen, Table 1), recovered after digestion with *Nsi*I/*Spe*I and cloned into a pSEC backbone purified from *Nsi*I/*Not*I-cut pSEC:Nuc [27] (Table 1). Since all primers were designed to eliminate first genes codons encoding for the native signal peptide (SP), in the resulting plasmids (pSEC:mTGF-β1 and pSEC:mSLPI), genes are fused in frame with a DNA fragment containing the ribosome binding site (RBS) and the SP of usp45 (SP_Usp45_), the gene encoding Usp45, the predominant *L. lactis*-secreted protein [28]. In these plasmids, the expression is controlled by the inducible promoter P_*nisA*_, the activity of which depends upon the concentration of nisin used [29]. Plasmids were introduced in *L. lactis* NZ9000 strain to obtain LL-mTGF-β1 and LL-mSLPI. Recombinant *L. lactis* strains expressing either murine IL-10 (LL-mIL-10) or human Elafine (LL-hElafin) have been previously reported [17].

**Table 1:** **Bacterial strains and plasmids used in this study**

**Strain or plasmid**	**Characteristic (s)** ^***a***^	**Reference or source**
	**Reference or source**	
**Strains**		
*E. coli* TG1	*supE, hsd, Δ5, thi, Δ(lac-proAB), F’(traD36 proAB-lacZΔM15)*	[23]
*L. lactis* MG1363	Wild type strain, plasmid free	[24]
*L. lactis* NZ9000	MG1363 (*nisRK* genes integrated into chromosome), plasmid free	[25]
*L. lactis* NZ9000 *htrA*	NZ9000 carrying *htrA* disruption (double-crossover recombination)	[22]
**Plasmids**		
pCR-TOPO	Ap^R^, subloning TOPO vector	Invitrogen
pORF9-mTGF-β1	Ap^R^, pORF9 plasmid carrying murine TGF-β1 gene	InvivoGen
pDK6-mSLPI	Ap^R^, pDK6 plasmid carrying full length murine secretory leukocyte protease inhibitor (SLPI) cDNA under the control of the MCMV promoter	[26]
pSEC:Nuc	Cm^R^, pGK plasmid (a derivative from the broad host range plasmid pWV01) expressing a secreted form of the staphylococcal nuclease (Nuc) under the control of P_*nisA*_ pomoter	[27]
pSEC:mIL-10	Cm^R^, pGK plasmid expressing a secreted form of murine IL-10 cytokine under the control of P_*nisA*_ promoter	[17]
pSEC:elafin	Cm^R^, pGK plasmid expressing a secreted form of human elafin antiprotease under the control of P_*nisA*_ pomoter	[17]
pSEC:mTGF-β	Cm^R^, pGK plasmid expressing a secreted form of murine TGF-β cytokine under the control of P_*nisA*_ pomoter	This study
pSEC: mSLPI	Cm^R^, pGK plasmid expressing a secreted form of murine SLPI antiprotease under the control of P_*nisA*_ pomoter	This study

^a^For strains, genotypic and phenotypic characteristics are given; for plasmids, plasmid and cloned-cassette characteristics are given.

### Nisin induction, protein samples preparation and immunoblotting

Recombinant *L. lactis* strains were grown to an optical density at 600 nm (OD_600_) of 0.6, followed by induction with 1 ng of nisin (SIGMA) per ml for 1 h as previously described [27]. *L. lactis* culture extraction and immunoblotting assays were performed as follows, using either murine SLPI (Santa Cruz Biotechnology, Inc., 10538), human Elafin (Santa Cruz Biotechnology, Inc., sc-20637) or murine IL-10 (Millipore, AB1492P) polyclonal antibodies. To determine SLPI, Elafin and IL-10 production, protein samples were prepared from 2 ml of induced cultures. After centrifugation (5 min, 10,000 *g*), the cell pellet (C) and supernatant (S) were treated separately. The S was treated with 100 μl of 100% trichloroacetic acid (TCA) to precipitate proteins and samples incubated for 10 min on ice. Proteins were recovered in PBS containing complete protease inhibitor cocktail tablets (Roche) after centrifugation at 4°C (10 min, 10,000 *g*). The C was treated by cell lysis in lysis buffer (25% sucrose, 1 mM EDTA, 50 mM Tris–HCl pH 8.0 and 10 mg/ml lysozyme) complemented with complete protease inhibitor cocktail tablets (Roche). Sodium dodecyl sulfate (SDS)-polyacrylamide gel electrophoresis, Western blotting, and immunodetection were performed following current protocol. The concentration of Elafin and IL-10 produced by recombinant lactococci was also assessed in both C and S samples by enzyme-linked immuno-absorbent assays: RayBio® human Trappin-2 ELISA Kit (RayBiotech, Inc. ELH-Trappin2-001) for Elafin and mouse IL-10 ELISA kit HRP (Mabtech, Sweden, 3431-1H-20).

TGF-β production and secretion by *L .lactis* was assessed by ELISA as described above using a human/mouse TGF-β 1 ELISA Ready-SET-Go kit (eBioscience, 88–8350).

### Preparation of live bacterial inocula for gavage of animals

RecLAB were grown as described above. Exponential growth cultures (OD600 = 0.4–0.6) of *L. lactis* strains were treated for 1–2 hour with nisin (1 ng/ml; Sigma) to induce recombinant protein expression. Recombinant strains of *L. lactis* were maintained with chloramphenicol (10 μg/ml). Bacteria were centrifuged after nisin induction, washed, and resuspended in corresponding volume of sterile PBS to get a final concentration of 5×10^9^ colony forming units (CFU) for intragastric administration (100 μl per mouse).

C57BL/6 mice (6–8 weeks old) were obtained from Janvier (Le Genest Saint Isle, France). All mice were kept at room temperature under 12 h light/dark cycles, and had free access to food and water. All procedures were approved by the Animal Care Committee “Midi-Pyrénées” (MP/06/12/02/12).

### Induction of colitis and study design

Colonic inflammation was induced by treatment with DSS (MP Biomedicals, Illkirch, France 160110), dissolved in drinking water (5% weight/volume). The animals were free to drink the DSS solution for 7 days. For the whole period of DSS exposure, mice were daily treated by intragastric gavage with 5×10^9^ CFU (in 100 μL PBS) of either wild-type (WT) or recombinant *L. lactis* strains or PBS (100 μL). Body weight was measured daily after the induction of colitis. On day 7 after adding DSS to their drinking water, mice were sacrificed and colons were analyzed and harvested for measurement of inflammation parameters: macroscopic and microscopic damage score, bowel thickness, myeloperoxydase (MPO) activity as previously described [14,17]. Upon sacrifice, the entire colon was excised and 1 ml PBS was instilled and washed twice through the lumen. Trypsin-like and elastase/PR-3-like activities were measured in those washes, using tosyl-Gly-Pro-Arg-*p*-nitroanilide (150 μM, Sigma) and MeO-succinyl-Ala-Ala-Pro-Val- *p*-nitroanilide (100 μM, Sigma) respectively as substrates. The change in absorbance at 405 nm was determined over 30-minutes at 37°C with a microplate reader NOVOstarTM (BMG Labtech, Champigny-sur-Marne, France). Activity was compared to known standard dilution of trypsin from porcine pancreas (Sigma) or human NE (Sigma) [14,17].

### Statistics

Data are presented as mean bars graph ± SEM. Statistical analysis were performed using one-way analysis of variance (ANOVA), followed by suitable post hoc comparison tests (Bonferroni’s or Dunnett’s). A *P* value less than 0.05 was considered significant. Graphic design and statistical analysis were performed using GraphPad Prism software version 5 for Windows.

## Discussion

The treatment of IBD represents one of the major challenges of modern medicine as they concern several millions of people. Current therapies for IBD strongly need to be improved since a high percentage of patients (20–40%) are resistant to any forms of treatments [30]. Moreover, severe side-effects and high costs are frequently associated to the currently available drugs (*e.g*. glucocorticoids and monoclonal antibody therapies).

Over the past 10 years, there has been increasing interest in the use of LAB as oral delivery vectors [4,7]. More particularly, the use of recombinant *L. lactis* secreting IL-10 for the prevention and treatment of colitis in different mouse models has been largely studied [11]. To date, two human clinical trials have been carried out with a biologically contained-derivative LAB (*ie*. lactic acid bacteria) secreting IL-10 [12]. The first study was performed in the Netherlands and assessed LAB IL-10 effects in Crohn’s disease patients. The results of this small phase I human trial, showed positive effects concerning biological containment, safety and tolerability [13]. In the second study (a phase IIa human clinical trial), ActoGenix (a company which develops genetically modified *L. lactis* for mucosal delivery of therapeutic molecules), assessed the effects of LAB IL-10 in subjects with moderately active ulcerative colitis. Unfortunately, although the results confirmed the suitability of the applied containment system in humans, no significant clinical effects were observed in this study (ActoGenix press release published at the end of 2009). This limited efficacy could be explained either by the fact that IL-10 is not the best molecule of choice to be used in a recLAB system, or by the fact that the IL-10 quantities delivered were not sufficient to be efficient in the intestinal environment.

The choice of IL-10 as a molecule to be delivered by LAB is supported by the anti-inflammatory effects of this cytokine [31]. As a matter of fact, mice deficient for IL-10 develop spontaneous colitis [32]. However, while systemic treatments with recombinant IL-10 are safe and well tolerated, they have a low therapeutic efficacy compared with placebo [33]. This suggests that pharmacodynamics of IL-10 are subtle and potentially needs a sustained and more mucosa-focused delivery to be efficient against colitis. Indeed, sustained delivery of IL-10 through adenovirus-based strategy showed better results in animal models [34,35], whether the colitis was induced by IL-10 deficiency or by chemicals. However, discrepancy exists on the fact that adenovirus-based IL-10 systemic delivery might not be able to reduce established colitis [36]. Local delivery of IL-10, through intracolonic administration of an adenovirus expressing IL-10 was able to reduce colitis in IL-10-deficient mice [37]. However, no study has investigated whether this mucosal delivery of IL-10 was efficient against colitis involving other mediators than only IL-10 deficiency. This approach of using LAB as vectors for IL-10 mucosal delivery [11], ensure a sustained and local (mucosal surface) delivery. However, IL-10 is released within the lumen, and might not be able to penetrate deep into the tissues and to exert its anti-inflammatory properties within mucosal tissues. IL-10 has broad immunoregulatory activity, acting to suppress intestinal inflammation on several levels. It inhibits T_H_1 lymphocyte differentiation; it diminishes antigen presentation and IL-12 release, and promotes differentiation and activity of regulatory T cells [31,38]. All these activities are related to actions on immune cells present in the *lamina propria*, not at the mucosal surface. Taken together, all these facts argue against considering IL-10 as a molecule of choice to be delivered by recLAB, and indeed, we demonstrated that treatments of colitic mice with IL-10 recLAB was not efficient on most parameters of intestinal inflammation. We cannot discard also that the minor effect observed with the LL-IL-10 strain was due to our murine colitis model. Indeed, acute colitis models induced by DSS are characterized by massive epithelial damage and it has been well established that IL-10 gave the best results in chronic colitis models that are immunological driven.

TGF-β1, another anti-inflammatory cytokine that could recapitulate some of the effects of IL-10 on immune cells, but that could also exert protective effects on intestinal epithelial cells, at the mucosal surface, appeared as a better candidate to be delivered by recLAB. Like IL-10, TGF-β1 exerts multiple actions on immune cells (regulatory T cells, Th17 cells, monocytes, macrophages) and is also protective in intestinal epithelial cells. Beck *et al*. have shown that TGF-β1 is required for intestinal mucosal healing, and strongly decreases the epithelial susceptibility to injury [39]. TGF-β1 also inhibits intestinal barrier disruption, by a direct effect on intestinal epithelial cells [40,41]. Thus, we constructed TGF-β1 recombinant *L. lactis* strain and tested this treatment against DSS-induced colitis. Like for IL-10 recombinant LAB, LL-TGF-β oral treatments were poorly effective against colitis. Such treatment only reduced granulocytes infiltration (Figure 2D), but not the other parameters of inflammation. Here again, one can wonder if TGF-β1 is the molecule of choice to be delivered by LAB, and could exert its anti-inflammatory effects, once released in the lumen. Therefore, we tested the effects of a completely different family of anti-inflammatory molecules, which are released by the intestinal epithelium, and at the mucosal surface: the serine protease inhibitors. Both Elafin and SLPI, when delivered by *L. lactis* and used as oral treatments, strongly inhibited DSS-induced colitis and were as effective (Figure 2). These serine protease inhibitors have different spectra of inhibition, SLPI being able to inhibit Cathepsin G and a number of trypsin like proteases (trypsin, tryptase and chymase), in addition to Elastase and Proteinase-3, also inhibited by Elafin [42,43]. This suggests that the sole inhibition of Elastase and Proteinase-3 is sufficient to obtain strong anti-inflammatory effects. Cathepsin G and trypsin-like inhibition should not provide additional protective effects. Treatments with recombinant *L. lactis* strains delivering either SLPI or Elafin were both more efficient to reduce signs of colitis, than treatments with anti-inflammatory cytokines recLAB. Endogenous protease inhibitors released physiologically by the intestinal epithelium thus appear as better molecules to be delivered by *L. lactis*, to generate potent therapeutic option. Indeed, these two protease inhibitors are pleiotropic in their role as guardian of mucosal surfaces. They not only inhibit the effects of proteases released by inflammatory cells, but they also inhibit pro-inflammatory transcription factors (AP-1, NF-κB), restore barrier functions, and exert antimicrobial activity [17,26,43,44]. Released by LAB in the lumen, Elafin and SLPI can potentially exert their anti-inflammatory biological functions more easily than anti-inflammatory cytokines. In addition, Elafin and SLPI might impact on the microbiota composition, due to their antimicrobial activity, might not be negligible. Such role could add to the protective effects they might have on the host [43].

An important point to consider when using the recLAB technology is the quantity of protein of interest that is delivered at the mucosal surface. Here, we showed that treatment with *L. lactis* strain inactivated in the main extracellular protease and thus able to release more protein of interest (Elafin), is more efficient at reducing colitis. Therefore, the recLAB technology could be improved by using such *L. lactis* mutant. The quantity of protein released at the mucosal surface could also depend on the time the bacteria will survive in the gastrointestinal tract (GIT). One of the hypotheses for the lack of effect of treatment with *L. lactis* IL-10 in clinical trials, was the fact that this bacterium has a weak resistance to the gastrointestinal environment [45]. Although our experiments were performed in mice and not in human like the clinical trials, we have observed that even with *L. lactis* as a vector, serine protease inhibitors were more potent anti-inflammatory molecules than IL-10 or TGF-β1. Nonetheless, other LAB vectors more persistent in the GIT (as lactobacilli) to deliver the proteins of interest should be tested to improve the kinetics and associated quantity of protein delivered at mucosal surfaces.

In conclusion, we have observed that the use of *L. lactis* strains expressing anti-proteases (Elafin or SLPI) is more efficient than the use of either LL-IL-10 or LL-TGF-β in this colitis model, to decrease intestinal inflammation. This differential effect could be explained by the different anti-inflammatory functions of these classes of molecules, and the fact that *L. lactis* vector delivery might be more appropriate with mucosal surface proteins such as protease inhibitors, than proteins exerting most of their effects in the *lamina propria*, such as anti-inflammatory cytokines. These results strongly suggest that the administration of recombinant *L. lactis* strains expressing serine protease inhibitors would raise more interest than the *L. lactis* IL-10 recombinant approach for IBD treatment.
